# Isopods Failed to Acclimate Their Thermal Sensitivity of Locomotor Performance during Predictable or Stochastic Cooling

**DOI:** 10.1371/journal.pone.0020905

**Published:** 2011-06-17

**Authors:** Matthew S. Schuler, Brandon S. Cooper, Jonathan J. Storm, Michael W. Sears, Michael J. Angilletta

**Affiliations:** 1 Department of Biology, Indiana State University, Terre Haute, Indiana, United States of America; 2 Department of Biology Bryn Mawr College, Bryn Mawr, Pennsylvania, United States of America; Institute of Marine Research, Norway

## Abstract

Most organisms experience environments that vary continuously over time, yet researchers generally study phenotypic responses to abrupt and sustained changes in environmental conditions. Gradual environmental changes, whether predictable or stochastic, might affect organisms differently than do abrupt changes. To explore this possibility, we exposed terrestrial isopods (*Porcellio scaber*) collected from a highly seasonal environment to four thermal treatments: (1) a constant 20°C; (2) a constant 10°C; (3) a steady decline from 20° to 10°C; and (4) a stochastic decline from 20° to 10°C that mimicked natural conditions during autumn. After 45 days, we measured thermal sensitivities of running speed and thermal tolerances (critical thermal maximum and chill-coma recovery time). Contrary to our expectation, thermal treatments did not affect the thermal sensitivity of locomotion; isopods from all treatments ran fastest at 33° to 34°C and achieved more than 80% of their maximal speed over a range of 10° to 11°C. Isopods exposed to a stochastic decline in temperature tolerated cold the best, and isopods exposed to a constant temperature of 20°C tolerated cold the worst. No significant variation in heat tolerance was observed among groups. Therefore, thermal sensitivity and heat tolerance failed to acclimate to any type of thermal change, whereas cold tolerance acclimated more during stochastic change than it did during abrupt change.

## Introduction

Organisms commonly modify their molecular and cellular structures to maintain performance as their environments change [1], [2]. Such acclimatory responses have been demonstrated to occur over temporal scales ranging from hours to months [3], [4]. For example, fruit flies can alter their thermal tolerance within the course of a single day [5], whereas trees require much longer to alter their photosynthetic rates [6]. When environmental conditions fluctuate slowly, an individual can continuously adjust its phenotype to match prevailing conditions (see [7]). In this way, organisms can tolerate variation in environmental conditions among seasons. Yet, some environments change rapidly and unpredictably, imposing costs for organisms that undergo acclimation [8]. When conditions fluctuate rapidly, the benefit of acclimation during an initial change could be offset by a loss of performance following a reversal [9]. Furthermore, stochastic variation weakens an individual's ability to anticipate future conditions and adjust its phenotype accordingly. These factors could explain why many organisms fail to acclimate to changes in their environment (reviewed by [10]).

Optimality models help researchers to explore how environmental fluctuations affect the evolution of acclimation. Gabriel [11], [12], [13] modeled reversible acclimation in an environment that switches between two states (e.g., hot and cold), whose conditions were described by a mean and variance. We can use Gabriel's model to generate hypotheses about thermal acclimation in a seasonal environment. The variance of environmental conditions in the model corresponds to uncertainty about the environmental temperatures during a seasonal shift. Based on this model, the selective pressure for thermal acclimation depends on the difference between seasons and the time lag for acclimation. Relatively large changes in temperature between seasons would select for genotypes with the potential to acclimate. Importantly, Gabriel assumed that the organism receives a reliable cue of environmental change, even though the precise magnitude of change remains unknown. In temperate environments, photoperiodic changes provide reliable cues to seasonal changes in temperature [14], [15]. Therefore, organisms from temperate regions should possess a marked capacity for thermal acclimation.

We studied the acclimation of thermal physiology in terrestrial isopods (*Porcellio scaber*) from the temperate environment of Terre Haute, Indiana, USA. In this location, isopods experience predictable variation among seasons and stochastic variation among days. In our experiment, we exposed isopods to abrupt, predictable, or stochastic changes in temperature and a predictable change in photoperiod. After this exposure, we compared their thermal sensitivities of running speed and tolerances of extreme temperatures. We expected that isopods would acclimate most readily when thermal cues were predictable. Because all isopods in our experiment came from the same selective environment, we expected variation in thermal physiology among treatment groups to stem primarily from the quality of thermal cues. Isopods exposed to constant and predictably declining temperatures received more reliable cues than did isopods expose to stochastically declining temperature. Thus, we predicted that thermal optima would vary among groups as follows: constant 20°C> stochastic decline > predictable decline > constant 10°C.

## Methods

### Study organism

The terrestrial isopod, *Porcellio scaber*, is widespread throughout Europe and North America, generally occurring within organic debris, leaf litter, and wood mulch. In urbanized areas, isopods are often found in cement cracks or seen moving across cement surfaces. In September of 2007, we collected 280 individuals from a suburban lot in Terre Haute, Indiana, USA. Each animal was weighed and placed in a Petri dish (90×20 mm) containing a thin layer of soil. Isopods were given pieces of carrot and potato twice a week. To prevent isopods from drowning, water was provided in the form of a gel (Cricket Quencher, Fluker Farms, Port Allen, LA). Petri dishes were misted with water 3–4 times a week to maintain a high humidity.

### Experimental design

**Figure 1:**
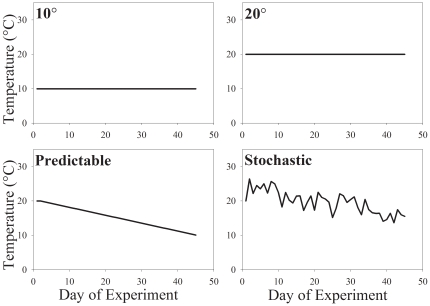
Four thermal treatments were used to study acclimatory responses by isopods: a stochastic decline in temperature that mimicked air temperatures in autumn; a predictable decline in temperature from 20°C to 10°C; a constant temperature of 20°C; and a constant temperature of 10°C.

We compared the thermal sensitivities and thermal tolerances among groups of isopods exposed to different thermal treatments for 45 days. Individuals were randomly assigned to either a constant temperature of 20°C, a constant temperature of 10°C, a predictable decline in temperature from 20° to 10°C, or a stochastic decline in temperature (Figure 1). Our constant thermal treatments approximated the means of the maximal and minimal daily air temperatures during the same period (20° and 10°C, respectively). The predictable decline in temperature consisted of a daily decrement of 0.2°C d^−1^ over the 45 days. The stochastic decline in temperature mimicked daily variation in air temperature recorded during October and November at a weather station in Terre Haute (Station 128723 of the National Climate Data Center, USA). These treatments enabled us to infer how isopods respond to different mean temperatures as well as to ecologically relevant declines in temperature. The photoperiod for each treatment shifted gradually from 11.8L:12.2D to 10.4L:13.6D over the course of the experiment. The changes in the light cycle mimicked the natural changes in sunrise and sunset for Terre Haute. Cycles of temperature and light were controlled by a programmable incubator (Model 818, Precision Scientific). Although spatial gradients of temperature within incubators were less than 1°C, Petri dishes were systematically rotated among shelves to eliminate any effect of thermal gradients on acclimation. We recorded the mass of each isopod before and after the thermal treatment.

After 45 days of exposure to the thermal treatments, we measured thermal sensitivities of running speed and tolerances of extreme temperatures. These measurements were completed within a period of 5 days. In between measurements, isopods remained in their respective thermal treatments; however, isopods in the declining thermal treatments experienced the same conditions as they did on day 45.

### Thermal sensitivity of locomotor performance

We measured the thermal sensitivity of running speed for 25 isopods from each thermal treatment. Speeds were measured on a narrow track (2×30 cm), with a rough surface and smooth walls (1 cm high). This track was kept in an environmental chamber that maintained the desired temperature. Each isopod was raced at six temperatures (8, 13, 20, 28, 32, and 36°C). The order of temperatures was determined randomly to avoid confounding temporal and thermal effects. Isopods were encouraged to run on the track by stroking their pleotelson with a camel-hair brush. Each individual was raced twice at each temperature; the greater speed was analyzed as the maximal performance. Although injuries rarely occurred, any isopod that sustained an injury during one of the trials was removed from the experiment.

### Critical thermal maximum

We estimated heat tolerance as the maximal temperature that enabled locomotion, usually referred to as the critical thermal maximum or knockdown temperature [16]. A subset of isopods from each thermal treatment, which were not subjected to previous measures of locomotor performance, were placed individually in small vials (10 mL). These vials were attached to a white sheet of plastic and were submerged in a water bath (Isotemp 228, Fisher Scientific) set at 38.0°C. We increased the temperature of the water by approximately 0.2°C per minute. The temperature was recorded when an isopod ceased to move its legs. At this time, we removed the vials from the bath for a few seconds to confirm the isopod could not respond to stimuli. Critical thermal maxima were measured for eight isopods at a time. Each trial included two isopods from each thermal treatment to avoid confounding effects of time and treatment.

### Chill-coma recovery

We estimated cold tolerance as the time required to recover from exposure to 0°C, usually referred to as chill-coma recovery [17]. A subset of isopods from each treatment, which were not subjected to measures of locomotor performance or heat tolerance, were placed in Petri dishes (50×10 mm). These dishes were entombed in ice, causing the air temperature within each dish fell to 0°C within 5 min. After 20 min, the dishes were removed from the ice and the isopods were transferred to sheets of paper at room temperature (21°C). Using a small brush, we positioned each isopod on its back in the center of a printed circle (diameter  = 20 mm). We recorded the time between the removal of dishes from the ice and the recovery of each individual using event-recording software [18]. Recovery was scored when an isopod assumed an upright position and broke the plane of the circle; this simple, objective measure of recovery reflected the onset of motor coordination [19]. As each isopod left its circle, we covered it with a small Petri dish to prevent the animal from interfering with others on the same sheet. Because isopods were assayed in successive trials, each trial included individuals from each of the four thermal treatments. Petri dishes containing isopods from different thermal treatments were chilled together, and the positions of these dishes were rotated between trials. To maximize our ability to detect and record recovery, no more than ten isopods were assayed at a time.

### Statistical analyses

We used an information-theoretic approach to evaluate several statistical models of the thermal sensitivities of running speed, typically referred to as performance curves [20]. Specifically, we used Akaike's information criterion (AIC) to compare the relative fits of five models: quadratic, Gaussian, modified Gaussian, exponentially modified Gaussian, and beta (Table 1). Models were fit to the data using the BFGS method [21] in the R Statistical Package [22]. When fitting the models, critical thermal maxima were used to estimate the upper thermal limits to performance. The model with the lowest value of AIC was used to compare performance curves among groups [23].

**Table 1:** A comparison of plausible models of the relationship between body temperature and running speed in isopods from four thermal treatments.

Treatment	Model	K	AIC	Δ*_i_*	Relative Likelihood	*w_i_*
10°C	Beta	6	152	0	1.000	0.952
	Gaussian	4	274	122	3.221·10^−27^	3.069·10^−27^
	Quadratic	4	286	134	7.985·10^−30^	7.606·10^−30^
	Mod. Gaussian	5	237	85	3.487·10^−19^	3.322·10^−19^
	Exp. Mod. Gaussian	6	158	6	0.049	0.047
20°C	Beta	6	164	0	1.000	0.993
	Gaussian	4	255	91	1.736·10^−20^	1.725·10^−20^
	Quadratic	4	249	85	3.487·10^−19^	3.464·10^−19^
	Mod. Gaussian	5	210	46	1.026·10^−10^	1.019·10^−10^
	Exp. Mod. Gaussian	6	174	10	0.006	0.006
Stochastic	Beta	6	273	0	1.000	0.970
	Gaussian	4	346	73	1.407·10^−16^	1.366·10^−16^
	Quadratic	4	351	78	1.155·10^−17^	1.121·10^−17^
	Mod. Gaussian	5	317	44	2.790·10^−10^	2.708·10^−10^
	Exp. Mod. Gaussian	6	280	7	0.030	0.029
Predictable	Beta	6	183	0	1.000	0.993
	Gaussian	4	264	81	2.577·10^−18^	2.560·10^−18^
	Quadratic	4	261	78	1.155·10^−17^	1.147·10^−17^
	Mod. Gaussian	5	229	46	1.026·10^−10^	1.019·10^−10^
	Exp. Mod. Gaussian	6	193	10	0.006	0.006

For all treatments, the beta model provided the best fit to the data. For each model, we report not only the AIC but also the differential AIC (Δ*_i_*), which is the difference between a given model's AIC and the lowest AIC. We also report the Akaike weight (*w_i_*), which is the normalized likelihood that the model is the best one in the set.

To compare thermal optima and performance breadths among groups, we used bootstrapping to generate confidence intervals for these parameters. For each group, data were sampled with replacement from the original set to create a new set with the same number of observations. Nonlinear models were fit to the resulting sets of data, as described above. For the model with the lowest value of AIC, we calculated the thermal optimum and the 80% performance breadth, (sensu [24]). Bootstrapping was performed a total of 10,000 times, which enabled us to compute confidence intervals for thermal optima and performance breadths (Table 2). These parameters were regarded as significantly different when no overlap existed between the 84% confidence intervals of the means for two groups, resulting in a Type 1 error rate of 5% [25].

**Table 2:** Thermal optima, performance breadths, and critical thermal maxima were similar for all treatment groups, but chill-coma recovery times varied significantly among groups.

Treatment	Thermal	Performance	Critical thermal	Chill-coma
	optimum (°C)	breadth (°C)	maximum (°C)	recovery (sec)
Constant 20°C	32.7 (31.8–34.3)	10.9 (9.3–13.2)	40.5 (40.1–40.9)	171 (113–276)
Stochastic decline	34.2 (32.5–35.2)	10.7 (8.3–12.1)	40.6 (40.3–40.9)	112 (101–140)
Predictable decline	33.5 (32.1–34.6)	11.0 (9.1–12.8)	40.6 (40.2–40.9)	129 (108–177)
Constant 10°C	34.4 (33.6–35.1)	10.0 (8.5–11.7)	40.4 (40.1–40.6)	130 (114–157)

Descriptive statistics are reported as means except for chill-coma recovery times, which are median values. Confidence intervals of the means are given in parentheses; 84% confidence intervals were calculated for means estimated by bootstrapping (thermal optima and performance breadths), and 95% confidence intervals were calculated for other means (critical thermal maxima and chill–coma recovery times).

As with thermal optima, we expected that the time to recover from chill-coma would vary among groups as follows: constant 20°C> stochastic decline > predictable decline > constant 10°C. To compare the mean chill-coma recoveries among treatment groups, we used an accelerated failure-time model fit to a Weibull distribution [26]. This model used a chi-square analysis to compare the expected recovery times for each treatment to the observed recovery times. Isopods that did not recover within one hour were censored in the analysis. The model was fit using the survival library of the R Statistical Package [22]. Median values are reported for the chill-coma recovery times, because the data were right-skewed (i.e., most individuals recovered rapidly).

## Results

**Figure 2:**
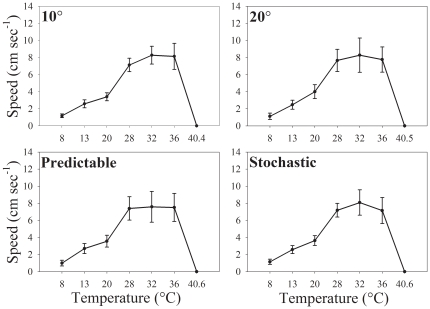
Performance curves were similar among isopod exposed to different thermal treatments. Labels for thermal treatments correspond to those used in Figure 1. No significant differences were observed among thermal optima or performance breadths for the four treatment groups (see Table 2). Error bars represent 95% confidence intervals.

Thermal sensitivities of running speed did not vary significantly among the four treatment groups (Figure 2). In all cases, a beta function provided the best fit to the data (Table 1). This superior fit likely resulted from the ability of the beta function to accommodate the skewed shapes of performance curves. Bootstrapping yielded very similar estimates of thermal optima and performance breadths for the groups (Table 2). Regardless of their thermal treatment, isopods ran fastest at 33° to 34°C. Likewise, all four curves were bounded by similar thermal maxima, ranging from 40.4 to 40.6°C (*F*_3,68_ = 0.39, *P* = 0.76; Table 2). Therefore, we failed to find evidence that the thermal sensitivity of running speed had acclimated to either constant or changing temperatures.

Some evidence of thermal acclimation was revealed by our comparison of cold tolerances. An accelerated failure-time model indicated that the time required for chill-coma recovery varied significantly among treatment groups (n = 109, *χ* ^2^ = 23.67, *P*<0.001). However, the rank order of recovery times differed from our hypothesis: constant 20°C> constant 10°C> predictable decline > stochastic decline (Table 2). Thus, isopods exposed to a stochastic decline in temperature tolerated cold the best and those exposed to a constant temperature of 20°C tolerated cold the worst.

## Discussion

We hypothesized that the thermal sensitivity of locomotor performance would change when isopods from a seasonal environment were exposed to naturalistic changes in temperature and photoperiod. Yet, isopods exposed to predictable and stochastic declines in temperature expressed thermal optima and performance breadths that were similar to those of isopods exposed to a constant temperature of either 10° or 20°C. Moreover, thermal optima were much greater than the mean environmental temperature of any treatment. Similar failures to adjust thermal physiology have been documented for other organisms exposed to changing environments. For example, a closely related species of isopods (*Porcellio laevis*) exhibited no change in the thermal sensitivity of rollover speed when exposed to thermal change [27]. Likewise, Niehaus and colleagues (in review) exposed field crickets to either constant or decreasing temperature, but observed no significant variation in the thermal sensitivities of feeding and locomotion. In contrast to our experiment, these studies did not include a treatment of abrupt thermal change (i.e., multiple constant temperatures). In our experiment, the absence of acclimation was unrelated to the pattern of thermal change (abrupt, gradual, or stochastic); in other words, isopods exposed to constant and fluctuating temperatures had similar thermal sensitivities.

Some species do alter their thermal sensitivity of locomotor performance during thermal change. In these cases, individuals usually display increased performance in a novel environment after a period of acclimation [28], [29], [30], [31]. Only rarely, however, does the thermal optimum of performance shift according to the mean environmental temperature. Such was the case in a recent study of the thermal acclimation of swimming speed in crocodiles [32]. Nevertheless, the capacity for thermal acclimation does not seem related to the magnitude and predictability of environmental variation. For example, genotypes from tropical and temperate environments often exhibit similar capacities for acclimation (reviewed by [10]). Furthermore, different species in the same environment exhibit markedly different capacities for acclimation. For example, Antarctic icefish (*Pagothenia borchgrevinki*) substantially altered their thermal breath of swimming performance when exposed to a warming of 5°C above natural conditions [33], whereas brittle stars (*Ophionotus victoriae*) were unable to tolerate a warming of 3°C [34]. Similarly, sea stars (*Odontaster validus*z) acclimated to 6°C [35], whereas other marine invertebrates from the same environment failed to acclimate to 3°C after two months of exposure [36], [37]. Even males and females of the same species differ in their ability to acclimate [38], [39]. As with our findings, this variation in the acclimation of thermal sensitivity cannot be explained by the current theory [11].

Variation in thermal tolerance generally makes more sense in light of the current theory [11], [13]. Heat and cold tolerances—as estimated by indices such as critical thermal maximum and chill-coma recovery—vary among populations and species along latitudinal clines (reviewed by [10], [40]). Studies of acclimation to constant or fluctuating temperatures suggest that natural variation in thermal tolerances partly stems from adaptation to local environments. For example, individuals exposed to high temperatures usually express higher thermal limits than do individuals exposed to low temperatures (e.g., [41]). In our study, the time required to recover from chill coma varied among groups in a way that partially supported our prediction. We expected that isopods that had been exposed to 10°C would recover the fastest, whereas isopods that had been exposed to 20°C would recover the slowest. As predicted, isopods exposed to 20°C took the longest to recover. Yet isopods exposed to 10°C did not recover faster than isopods exposed to either predictable or stochastic declines in temperature. Interestingly, this variation in cold tolerance was not associated with variation in heat tolerance, which accords with patterns observed in other species [42], [43].

Although few studies have included thermal fluctuations, we can conclude that the acclimation of thermal tolerance does not necessarily depend on the variance of environmental temperature. Support for this idea comes from a recent study of zebrafish (*Danio rerio*); Schaefer and colleagues [44] found that fish exposed to warm conditions, whether constant or fluctuating, had higher critical thermal maxima than did fish exposed to cool conditions. That said, the strength of the interaction between the mean and variance of temperature likely depends on the range of values chosen for these parameters [45], [46]. Individuals exposed to high mean temperatures and high variances are most likely to experience selection for heat tolerance, whereas those experiencing low mean temperatures and high variance are most likely to experience selection for cold tolerance. Such interactions would demand the use of realistic thermal fluctuations if biologists wish to draw ecological inferences from laboratory experiments.

Unlike most studies of acclimation, our experiment involved a gradual shift in photoperiod in addition to several patterns of thermal change. Gradual changes in photoperiod provide reliable cues about seasonal changes in temperature (reviewed by [14]), and thus should facilitate thermal acclimation. To separate thermal and photoperiodic cues, we exposed all four groups of isopods to the same change in photoperiod while exposing each group to a different change in temperature. Thus, any variation in thermal sensitivity or thermal tolerance among the groups must have been caused by differences in thermal cues. Since we observed no variation in thermal sensitivity among groups, we concluded that changes in temperature did not trigger the acclimation of locomotor performance. However, we cannot know whether the identical shift in photoperiod throughout the experiment caused the thermal sensitivities of isopods in all groups to acclimate similarly. In other words, thermal acclimation of isopods might be triggered completely by photoperiod, a mechanism that could only be detected by comparing groups exposed to different photoperiods. Strong photoperiodic control of thermal acclimation has been observed in some ectotherms, such as fruit flies (*Drosophila spp.*) [5] and rainbow trout (*Oncorhynchus mykiss*) [47]. Interestingly, other studies have documented thermal acclimation under a constant photoperiod [48], [49]. If photoperiod controlled thermal acclimation in our experiment, we should still wonder why the thermal optimum of locomotion was much higher than the temperatures experienced by the isopods. Moreover, isopods ran poorly at all temperatures included in our thermal treatments (see Figure 2), suggesting that acclimation of thermal breadth had not occurred either. Perhaps more will be learned by combining realistic thermal and photoperiodic cues when comparing the acclimatory responses of genotypes from different environments.
